# Cognitive States Matter: Design Guidelines for Driving Situation Awareness in Smart Vehicles

**DOI:** 10.3390/s20102978

**Published:** 2020-05-24

**Authors:** Daehee Park, Wan Chul Yoon, Uichin Lee

**Affiliations:** Graduate School of Knowledge Service Engineering, Korea Advanced Institute of Science and Technology, 291, Daehak-ro, Yuseong-gu, Daejeon 34141, Korea; daehee.park@kaist.ac.kr (D.P.); wcyoon@kaist.ac.kr (W.C.Y.)

**Keywords:** cognitive engineering, situation awareness, situation awareness design guidelines

## Abstract

Situation awareness (SA) is crucial for safe driving. It is all about perception, comprehension of current situations and projection of the future status. It is demanding for drivers to constantly maintain SA by checking for potential hazards while performing the primary driving tasks. As vehicles in the future will be equipped with more sensors, it is likely that an SA aiding system will present complex situational information to drivers. Although drivers have difficulty to process a variety of complex situational information due to limited cognitive capabilities and perceive the information differently depending upon their cognitive states, the well-known SA design principles by Endsley only provide general guidelines. The principles lack detailed guidelines for dealing with limited human cognitive capabilities. Cognitive capability is a mental capability including planning, complex idea comprehension, and learning from experience. A cognitive state can be regarded as a condition of being (e.g., the state of being aware of the situation). In this paper, we investigate the key cognitive attributes related to SA in driving contexts (i.e., attention focus, mental model, workload, and memory). Endsley proposed that those key cognitive attributes are the main factors that influence SA. In those with higher levels of attributes, we found eight cognitive states which mainly influence a human driver in achieving SA. These are the focused attention state, inattentional blindness state, unfamiliar situation state, familiar situation state, insufficient mental resource state, sufficient mental resource state, high time pressure state, and low time pressure state. We then propose cognitive state aware SA design guidelines that can help designers to effectively convey situation information to drivers. As a case study, we demonstrated the usefulness of our cognitive state aware SA design guidelines by conducting controlled experiments where an existing SA interface is compared with a new SA interface designed following the key guidelines. We used the Situation Awareness Global Assessment Technique (SAGAT) and Decision-Making Questionnaire (DMQ) to measure the SA and decision-making style scores, respectively. Our results show that the new guidelines allowed participants to achieve significantly higher SA and exhibit better decision making performance.

## 1. Introduction

Situation awareness (SA) is about recognizing and understanding current situations and predicting possible changes in the future [1]. In vehicular contexts, drivers need to constantly maintain SA for safe driving. For example, drivers should attend to their route location, the shape of the road ahead, nearby traffic, signs and signals, unexpected hazards, the state of their vehicle, and how all these things are changing moment by moment [2]. Failure to maintain SA or poor SA negatively influences driving safety [3]. Thus, drivers should keep themselves updated about their current situation, which allows them to create projections about future situations.

Constantly maintaining SA is challenging because driving situations are dynamically changing. Automotive manufacturers have been building smart cars equipped with advanced aiding systems that connect to various sensors regarding situation information and help drivers to maintain SA. Furthermore, recent advances in on-board sensing and machine cognition will greatly diversify situation information ranging from local and remote road contexts to short- and long-term contextual changes. Due to limited human cognitive capabilities, however, drivers cannot accept many kinds of complex situation information while driving. Gottfredson suggested that cognitive capability can be defined as a general mental capability that includes planning, complex idea comprehension, abstract thinking and learning from experience [4]. Thus, the method of presenting situation information is important in the context of driving smart cars.

**Figure 1:**
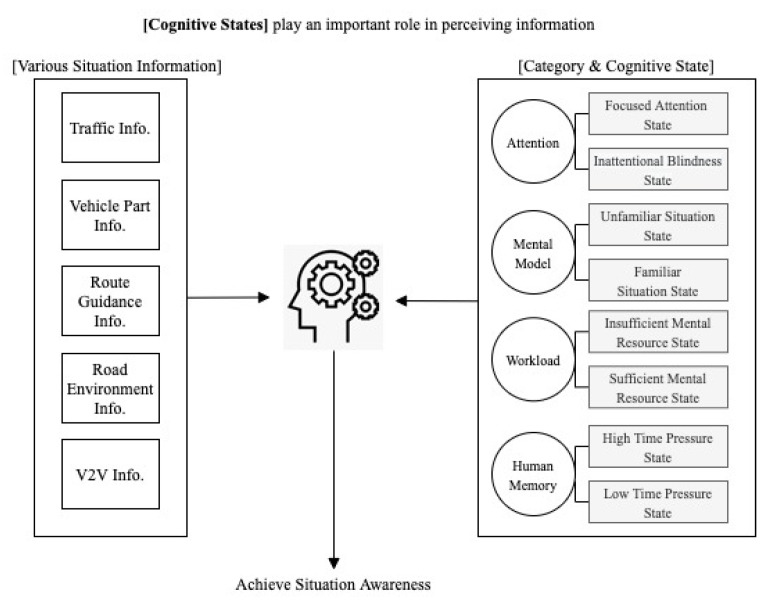
Cognitive states play an important role in perceiving situation information and in achieving situation awareness (Source: Own creation).

Endsley developed high-level SA design principles for creating SA aiding systems that aim to enhance the driver’s SA (e.g., organize information around goals and support comprehension) [3]. However, Endsley’s SA principles offer only high-level insights for system design and lack detailed guidelines on how to consider human cognitive states, their related SA errors and causes, and countermeasures. There is a consensus that cognitive processes and activities underlie achieving and maintaining SA [5,6], including attention (divided and focused), memory (short- and long-term), perception (pattern recognition/matching, visual search, and processing speed), and executive control [7]. Lyu et al. further showed that dynamically changing driving situations may affect such cognitive states, which in turn influences driving performance [8]. Advanced aiding systems can mitigate this problem by offering better situation awareness, but delivering diverse and complex situation information may overload drivers’ cognitive capacities, leading to human errors and accidents. The driver could perceive and interpret the information differently depending on the cognitive states [8] (Figure 1). A cognitive state is generally defined as a condition of being (e.g., the state of being alert, the state of being aware of the situation, the state of being certain or uncertain and the state of knowing something) [9]. Thus, we develop a set of supplementary SA design guidelines that consider diverse cognitive states and demonstrate their usefulness through a case study.

This paper is organized as follows. We reviewed the definition of SA and the design principles suggested by Endsley (Section 2). We considered the causes of SA errors, reviewed Endsley’s SA design principles, and justified the need for supplementary SA design guidelines (Section 2). In addition, we described how we developed the new SA design guidelines (Section 2). Then, we depicted how we designed the case study to validate the guidelines (Section 2). We proposed cognition-aware SA design guidelines based on the human driver’s cognitive state to help designers to consider a driver’s cognitive states (Section 3). The guidelines have been developed based on the following eight cognitive states which play an important role in achieving SA: focused attention state, inattentional blindness state, unfamiliar situation state, familiar situation state, insufficient mental resource state, sufficient mental resource state, high time pressure state, and low time pressure state. Our SA guidelines focus on the key cognitive states (which are categorized as attention, memory, mental model, and workload) and their expected SA errors. Finally, to demonstrate the helpfulness of the proposed guidelines, we designed a new SA aiding system following our guidelines and conducted a case study that compared the current and revised telematics systems (Section 4). We measured the situation awareness and decision making style scores by using the Situation Awareness Global Assessment Technique (SAGAT) and Decision Making Questionnaire (DMQ), respectively [3,10]. These methods are widely used and have been validated to measure the SA and decision making style scores [3,10]. Our results showed that our new SA aiding system achieved higher SA and promoted better decision-making (Section 5).

## 2. Methods

### 2.1. Background

#### 2.1.1. Situation Awareness and Human Errors

We reviewed the definition of SA, the types of human errors caused by SA, and Endsley’s SA principles. Endsley showed that situation awareness is a prerequisite for decision-making and action, and it can be divided into three levels: perception, comprehension, and projection [5].

Perception (SA Level 1): The driver is required to perceive the status, attributes, and relevant information in the environment [5]. According to Jones and Endsley, a level 1 error is the failure to correctly perceive the situation [11]. Level 1 perception errors comprised approximately 76% of SA errors in the aviation domain [11]. The causes of level 1 errors are generally simple omission, attentional narrowing, distraction, and high workload [12]. There are two more possible causes. The information may be directly available but not observed or included in the scan pattern [5], or the needed information may not be provided clearly to the driver [3]. In addition, if the method of presenting information is wrong, then the driver cannot perceive it [13].

Comprehension (SA Level 2): The driver comprehends the meaning of the information [5]. A level 2 error is the failure to comprehend the situation. According to Endsley, comprehension errors occur when the information is correctly perceived, but its meaning is not comprehended [5]. In addition, level 2 errors include the failure to comprehend the cause and meaning of information [13]. Level 2 errors comprised approximately 19% of SA errors in the aviation domain [11]. The causes of level 2 errors include having an incorrect mental model, being unfamiliar with the situation, and having limited working memory [12]. In addition, the use of incorrect mental models can also be caused by level 2 errors. Regarding the use of incorrect mental models, although the driver has a good model of how a system functions, there is an over-reliance on default values in the model [5]. These defaults can be thought of as general expectations about previous information that have not been updated in real-time. For example, although there is a new route, the driver’s mental model may not be updated with the new route. Thus, he may use the old route information to guess the driving time. In addition, the driver sometimes does not comprehend the importance of perceived information related to goals, and this may be due to working memory limitations [12].

Projection (SA Level 3): Level 3 SA occurs when the driver anticipates what will happen in the future after perceiving the information and understanding the meaning of the information [5]. The driver can obtain level 3 SA when the driver has a deep comprehension of the situation. Projection allows the driver to perform the task by proactively making decisions. If the driver achieves a clear projection, it helps the driver make an easy and effective decision. A level 3 error is the failure to project situations into the future. Level 3 projection errors comprised approximately 6% of SA errors in the aviation domain [11]. Level 3 errors are caused when the driver is fully aware of what is going on in the present but has a poor mental model for projecting what that means for the future [5]. In addition, the main reason that level 3 SA errors occur is insufficient mental resources [3].

**Figure 2:**
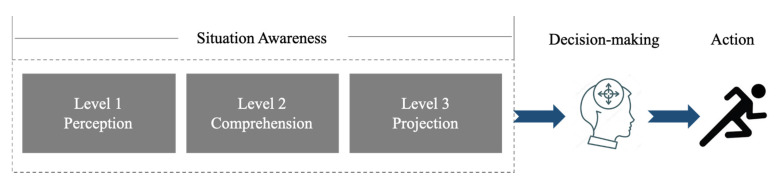
A simple version of Endsley’s model: situation awareness (SA), decision-making, and action (Own Creation).

Decision-Making and Action (After SA): Achieving SA affects decision making and action (Figure 2). A judgment about whether the driver has good SA or not can be made based on the driver’s actions [5].

#### 2.1.2. Endsley’s SA Design Principles

Endsley suggested the concept of Situation Awareness Oriented Design (SAOD) as a design method [3]. Endsley emphasized the importance of the SA system interface design to present the proper situation information to the operator at the right time without cognitive burden [3]. Thus, Endsley developed general SA design principles to help the designer understand SAOD, and the designer can apply them to the design of an SAOD system. Table 1 describes Endsley’s SA principles [3].

The first principle, “Organize information around goals”, says that information should be organized according to the main objectives of the operator rather than expressed in a technology-oriented manner [3]. “Present Level 2 information directly—support comprehension” reflects the fact that the degree to which integrated information satisfies level 2 SA will positively influence SA [3]. “Provide assistance for level 3 SA projections” is related to the fact that the operator is required to develop a proper mental model to make projections about future states [3]. “Support global SA” means that the operator should have a high-level overview of the situation related to the goals [3]. “Support trade-offs between goal-driven and data-driven processing” deals with the tension between top-down processing and bottom-up processing when designing a system. The system should support both top-down processing (principle 1) and bottom-up processing (principle 2), and these modalities should complement each other [3]. “Make critical cues for schema activation salient” aims to activate cognitive functions by offering saliency in user interactions [3]. “Take advantage of parallel processing capabilities” refers to the fact that the operator can share attention in SA [3]. “Use information filtering carefully” means that cognitive overload could hinder situation awareness, and filtering can lower the cognitive workload [3].

### 2.2. Design Guideline Development

#### 2.2.1. Limitations of Ensley’s SA Principles for Vehicular SA Design

We discuss several limitations of Endsley’s SA principles that do not make them easily applicable to driving contexts.

First, it is not clear how existing SA principles can be applied based on human cognitive states. Situation information could be complex, and thus, the amount of information will vary depends on the situation. For example, in the case of driving strategy information, “Support global SA” can be applied. However, in the case of traffic information about a lane, “Provide assistance for level 3 SA projections.” can be applied. The quantity of information and period of the presenting situation will vary, and the level of information acceptance also will vary depending on the driver’s cognitive state. Cognitive processes and activities change according to the situation, and changes to cognitive processes affect the driver’s cognitive state. In addition, the driver’s ability to accept the situation information varies depending on the cognitive state. For example, when the driver is in an insufficient mental resource state while driving, the driver cannot have global SA due to very limited cognition capability, whereas in a sufficient mental resource state, the driver can easily have global SA.

Second, existing SA principles do not discuss the SA errors that occur in different cognitive states. The designer should first understand the expected SA errors and causes; then the designer can choose which SA principle to apply. Several studies have identified various human cognitive states that are intertwined with SA related errors [14,15,16]. Difficulty in recognizing SA errors and causes several principles to in conflict with each other. Pesavento insisted that sometimes several principles conflicted, such as “Support global SA.” and “Organize information around goals”. [17] Supporting global SA means presenting more subset information to help the operator understand all related environments, whereas organizing information around goals means that the information displayed should be organized around the operator’s goal, rather than according to a technology-driven approach. However, the driver’s goals are always changing according to the situation. Sometimes, the driver needs global SA to check all driving environments, but sometimes, the driver only needs a small amount of information that is applicable to the goals of the given situation. Thus, when applying Endsley’s SA principles to diverse cognitive states, the expected SA errors should be considered.

Third, existing SA principles do not consider the countermeasures corresponding to expected SA errors that occur in certain cognitive states. Pesavento also insisted that some principles failed to direct the designer in a particular direction [17]. Some of Endsley’s principles, such as “Present Level 2 information directly—support comprehension” and “Provide assistance for level 3 SA projections”, do not explain how to present level 2 information directly or how to provide assistance for level 3 projections [17]. In the case of “Make critical cues for schema activation salient” (Endsley’s SA principle), it is difficult to make design decisions about how to present the cue [17]. Thus, we believe that detailed guidelines about countermeasures that correspond to SA errors and causes should be added to help the designer to know how to easily apply Endsley’s SA principles.

#### 2.2.2. Design Guideline Development Methods

In order to determine the relationship between human cognitive states and SA, we reviewed existing literature about human cognitive states, especially those that cause people to have difficulty achieving SA in dynamic situations. We describe ways in which we developed the new supplementary SA design guidelines. First, we explain how we have identified target cognitive states and also each cognitive state in detail, with a focus on the aspects of the definition that are related to the SA guidelines. In order to determine the relationship between human cognitive states and SA, we reviewed existing literature on human cognitive states, especially those that cause people to have difficulty achieving SA in dynamic situations. According to the human information processing model (Wickens et al.), the working memory and long-term memory influence the perception ability, and this is connected to decision and response selection [14]. Endsley proposed that attention, mental model, workload, and memory mainly influence SA [15]. Thus, we added supplementary design guidelines in four categories: attention, mental model, workload, and human memory. Then, we chose eight cognitive states which can occur under the above-mentioned four categories while driving. Second, we describe the cognitive state-awareness guidelines.

These guidelines include the main SA problems in each cognitive state and guidelines on ways to respond to the SA problems. To develop the guidelines, we used a theoretical analysis which is similar to the method of developing SA principles proposed by Endsley [3]. First, we defined several human cognitive states that could occur during driving. Second, we added expected SA problems based on each cognitive state. Third, we suggested corresponding guidelines.

### 2.3. Case Study Design

The cognitive state-aware SA guidelines were supplemented based on human cognitive states for safe driving. We believed that applying these guidelines to the SA aiding system would influence the way that complex situation information was presented to the driver. Hence, we conducted a case study to understand the effectiveness of our new cognitive state-aware SA design guidelines, and to demonstrate how to apply the new SA design guidelines to an SA aiding system. Teegavarapu et al. suggested that case studies should be used in design research to analyses a phenomenon, generate hypotheses, and validate a method [18]. According to Abercrombie, the case study is useful in the preliminary stages of an investigation [19].

The purpose of this case study was to demonstrate the validity of the cognitive state-aware SA design guidelines by building a new SA aiding system. The research question was to determine whether our new way of presenting information was more efficient than the current telematics system under diverse driving contexts. The hypothesis of the case study was that the new SA aiding system helps the driver to obtain higher SA and leads to effective decision-making. To show the effectiveness of the SA aiding system, we measured SA and decision-making style scores. Further, the guidelines were applied to the aiding system to help the driver to enhance SA and to conduct effective decision-making while driving. The cognitive state affects the level of SA, and the driver’s level of SA also influences decision-making and action. We decided to measure the driver’s decision-making abilities, which can be regarded as a result of SA after the situation information has been given. We used Situation Awareness Global Assessment Technique (SAGAT) and Decision Making Questionnaire (DMQ) to measure the SA and decision-making style scores for two driving scenarios. These scenarios were developed to incorporate three cognitive states. The techniques of SAGAT and DMQ have been validated as effective methods to measure SA and decision-making style scores [3,10].

In our current evaluation, we have chosen three cognitive states (i.e., insufficient mental resource state, unfamiliar situation state, and high time pressure state). These dimensions can significantly affect SA when compared to the other dimensions, which were excluded for the following reasons. The focused attention state and inattentional blind state are quite difficult to reproduce during driving simulations, and the level of these states could also be quite different for different individuals. There are also less concerning cognitive states such as the familiar situation state, sufficient mental resources state, and low time pressure state where drivers are less likely to have an incorrect or poor mental model, and reproducibility of participants’ individual mental models would be low.

#### 2.3.1. Metrics

We measured situation awareness score and decision making style score by using situation awareness global assessment technique (SAGAT) and decision making questionnaire (DMQ).

##### SAGAT

In order to measure SA, we used SAGAT, which is a global tool that was developed to assess all elements of SA using a comprehensive assessment of driver SA requirements [5]. SAGAT has been widely used and proven to be effective in various domains such as aviation, air traffic control, and driving [5]. SAGAT helps the experimenter to measure each level of SA during the specific driving scenario. As SAGAT provides a global measure, it involves questions regarding the requirements of SA. Thus, the questions cover level 1 SA for ‘Perception’, level 2 SA for ‘Comprehension’, and level 3 SA for ‘Projection’. While conducting SAGAT, simulations of interest in the system can be paused at randomly selected times, the system display can be blanked, and then, the participants can be asked to answer SA questions at that time.

##### DMQ

In order to measure how the driver makes a decision, we used the DMQ, which is a questionnaire developed by French et al. to measure decision making style in different dimensions [10]. The DMQ allows the experimenter to analyses the decision-making style in various dimensions. In this case study, only those dimensions relevant to SA were chosen: control, thoroughness, and hesitancy.

Control: If the driver recognizes, understands, and predicts what will happen in the future, the driver can control the situation itself, and if it is easy to control the situation, decision making will be efficient.

Thoroughness: If the driver is thorough about the situation, he might have a good understanding of the situation, have a proper mental model, and be able to make efficient decisions.

Hesitancy: If the driver can accept sufficient situation information, he will not hesitate to make a decision because he can easily recall the relevant knowledge from long-term memory, and he can easily understand the situation and predict what will happen in the future. On the other hand, misunderstanding the situation induces a delay in decision making when the driver needs to act since the driver cannot control the whole situation. In order to measure how each condition influences delays in the driver’s decision making, we measured “Hesitancy”.

#### 2.3.2. Prototype Development Methods

We developed the current telematics system and the new SA aiding prototype for the case study through a prototyping application called Flinto [20]. It is a UI prototype, and its functions are limited. Overall, we constructed a simulated driving environment through a simulator system called SCANeR Studio version 1.8 [21]. Our prototype presents the SA information and support interactions according to the pre-defined driving scenarios. We used the method of the Wizard of Oz to conduct the experiment.

## 3. Cognitive State-Aware SA Guidelines for the Driving Environment

### 3.1. Major Cognitive States Categories

Attention: The way in which the driver deploys his attention in acquiring and processing information influences SA. The focused attention state and inattentional blindness state were chosen as human cognition states while driving. Those attentional states are related to working memory and influence perception. A driver in the focused attention state may lack the global SA required to formulate and revise his/her driving strategy [3]. Attentional focus is a salient cognitive state and an important potential source of perceptual error [3]. Whether intentionally or unintentionally, the driver’s attention can sometimes become too focused when performing a task, and as a result, they may fail to perceive the relevant stimulus. In the inattentional blindness state, the driver cannot perceive or understand salient cues or alerts even though the driver sees them [14]. Wickens et al. reported that inattentional blindness means that although there may be sufficient attentional capacity to maintain primary task performance, the driver may not notice unexpected events [14].

Mental model: A mental model helps the driver to determine what information is critical to pay attention to and from expectations [3]. Mental models have been succinctly defined as “mechanisms whereby humans are able to generate descriptions of system purpose and form, explanations of system functioning and observed system states, and predictions of future states” [22]. The unfamiliar situation state was chosen because if the driver encounters an unfamiliar situation or the driver is a novice, then the driver does not have a proper mental model. Endsley suggested that when the driver encounters a new type of situation or if the driver is a novice, he/she may not have an appropriate mental model of the situation to derive, and thus, he may be at a disadvantage when developing level 2 SA [3]. This influences level 2 comprehension [3]. The familiar situation state was also chosen because there is a possibility of failure in comprehension. A familiar situation state occurs when the driver is an expert, or when the driver encounters a familiar situation. Even in a familiar situation state, the driver may commit human behavioral errors. Although the driver has a good model, there is over-reliance on the old version of the information in the model [5].

Workload: Workload refers to the amount of work that the driver can perform at a certain time, the amount of information the driver can process, or the mental effort required to complete a task [23]. Workload and SA are intricately intertwined [24]. According to Endsley, when the driver has insufficient mental resources, this is a reason for failure to accurately project the system state from a level 2 SA [3]. When the driver has a higher workload, it induces the driver’s cognitive state to change to an insufficient mental resource state since the driver only processes a limited amount of information at a time [3]. According to Endsley, lack of mental resources is a reason for the failure to accurately project the system state from a level 2 SA [3]. An insufficient mental resource state occurs when the driver’s mental workload is high. The sufficient mental resource state can also be regarded as a normal state during driving. However, since the driver is focusing on performing primary tasks, the driver’s workload may be increased depending on the amount of information given at a time and the timing of presenting the information. The sufficient mental resource state occurs when the driver’s mental workload is low. Although the driver has sufficient mental resources to perceive the situation, the driver’s workload may be increased depending on the amount of information given at a time and the time at which the information is presented since the driver is focusing on performing the primary tasks. This leads the driver to enter into an insufficient mental resource state.

Human memory: Working memory plays a fundamental role in helping to achieve SA [3]. Regarding working memory, the time pressure state was chosen as a human cognitive state since it affects working memory and the ability of the driver to comprehend the situation. Chuderski suggested that time pressure blocks complex cognitive processes and working memory [16], and high time pressure has been shown to reduce the quality of decision making [25]. In a high time pressure state, the driver may have difficulty analyzing all relevant information before making a decision since working memory is limited. Endsley insisted that time plays an important role in levels 2 and 3 SA [3]. The time pressure state occurs when the driver has to complete the most pressing tasks in a limited time, and while completing the tasks, the driver reacts to the time pressure by narrowing down the number of alternatives [26]. Alternatively, a low time pressure state can be regarded as a normal state during driving. However, since the driver is focusing on performing the primary tasks, the driver may enter a high time pressure state if the transfer of information is delayed.

### 3.2. Cognitive State-Aware SA Guidelines

We developed detail SA guidelines in four categories. The SA guidelines based on cognitive states are organized as shown below (Table 2).

To present information to a driver in this cognitive state, the new SA guidelines state that the SA aiding system should provide global SA information related to the driving strategy to the destination to facilitate effective driving. Presenting detailed situational information is regarded as an effective way to expand the SA of the whole environment [3]. Because the driver is focused on performing the primary tasks, if the information given is related to the driving strategy to the destination, the driver can drive more effectively and make decisions such as changing the route, changing to a faster lane, or using a safer road.

- Inattentional Blindness State

If the driver is in a state of inattentional blindness, the driver cannot easily perceive new information. To present information to a driver in this cognitive state, the new SA guidelines state that the system should use overt communication to get the driver’s attention because the driver should be required to focus on the primary tasks. According to Sperber and Wilson, overt communication is a way of communicating with a listener with the informative and communicative intention [27]. It is an obvious and noticeable method of communication [27]. The system can divert the driver’s attention from performing the primary tasks to present situational information since overt communication works as a critical cue. If a driver is in the inattentional blindness state, the SA aiding system should help the driver to refocus his/her attention using overt communication even if doing so interferes with the task to a slight extent. Although overt communication may interfere somewhat with the driver’s task, using it to convey relevant information will align the driver’s attention with the aiding system to avoid perception errors.

- Unfamiliar Situation State

When the driver is in an unfamiliar situation state, the driver cannot understand the cue or alert even if he sees it (comprehension error). Thus, understanding, projection, decision-making, and action are relatively slow or inappropriate in an unfamiliar situation because the driver lacks an appropriate mental model, and it takes time to retrieve similar experiences or knowledge.

To present the information in this cognitive state, the new SA guidelines state that, first, the system should present information proactively to refocus the driver’s attention to the extent that it does not interfere with the driver’s primary task, giving them time to recall the proper knowledge to update his mental model and respond to the situation. Novices would be less familiar with hazardous situations and so would be required to deduce the danger in an effortful manner instead of simply recognizing it [28]. When the system presents information, it should not interfere with the driver’s primary tasks. The multiple resource theory asserts that people have a limited set of resources available for mental processes [29]. Multiple resource theory as described by Wickens says that multiple attentional resources exist and, in some cases, are separate from one another [30]. It is suggested that people can perform different attentional tasks at the same time without interference. However, the workload is increased when additional resources are needed for performance [30]. Thus, the system should present information to the extent that it does not interfere with the driver’s primary task.

Second, the system divides a large amount of information and provides it to the driver little by little to give the driver sufficient time and help the driver to understand the actions he/she needs to perform.

- Familiar Situation State

This causes the driver to create fewer projections for future situations. That is the reason why we also have chosen a familiar situation state. For example, when the driver does not use the navigation system because of familiarity with the route, the driver’s assumption about the time to the destination might be wrong due to unexpected heavy traffic. Thus, the driver might not be aware of traffic info.

To present information to drivers in this cognitive state, the new SA guidelines state that, first, the presented information needs to help the driver update their mental model with new information to avoid over-reliance on the old version of the information in the mental model and to make predictions about future situations. Second, the SA aiding system should indicate appropriate actions sequentially at the appropriate time to help the driver execute these actions. When a driver performs an action, the system should divide the action into detailed tasks and guide the driver at appropriate times to avoid mistakes due to the existing mental model.

- Insufficient Mental Resources State

The insufficient mental resources state is associated with not only projection but also perception, comprehension, and decision-making. Although some SA is present, the insufficient mental resource state can affect other elements of SA since the driver is not able to retrieve the proper knowledge from memory. For example, the driver’s workload is high because several cognitive tasks must be performed concurrently using a limited amount of cognitive resources.

To present information to a driver in this cognitive state, the new SA guidelines state that, to avoid increasing the driver’s workload, the system should priorities and summaries information that is salient for direct perception, and then provide this information to the driver to the extent that it does not interfere with the driver’s primary task. When the driver is in an insufficient mental resource state, the driver cannot accept large amounts of information at a time due to a high workload. Thus, the driver should receive a small amount of information, and this should help the driver to project what will happen in the immediate future. Direct perception means that ‘people directly perceived the higher order variables that the ecology had to offer them, without any mediating information processing’. [31].

- Sufficient Mental Resources State

In this state, the driver makes SA errors at all three levels. For example, when the driver is in a sufficient mental resources state, if the driver does not understand the information or the information is too complex, it can increase the driver’s workload.

To present information to a driver in this cognitive state, the new SA guidelines describe that, first, the system should present more detailed information to enhance Level 2 and Level 3 SA, but that information should be configured and provided to avoid increasing the workload too much. Because the driver’s mental workload is low, the driver is able to consider the situation in detail to understand it correctly. The information provided by the system should be provided in detail within a range it does not increase the driver’s workload too much. Second, the system should emphasize information that needs to be known in the moment the extent that it does not interfere with the driver’s primary task. According to Theeuwes, the driver’s workload is increased when the driver feels situations are unclear such as when there is unclear signing, and in situations where it is unclear what behavior is required [32]. After information prioritization and summarization, the system can emphasize the information that needs to be recognized in the moment to avoid increasing the workload.

- High Time Pressure State

In high time pressure state, there is a risk that mistakes will be committed due to the urgency of the task (errors of perception, comprehension, projection, decision, and action. For example, when the driver needs to arrive at a destination at a fixed time, the driver cannot control the whole situation. As a result, lane keeping accuracy may be poor, and the driver may break the speed limit [33].

To present information to a driver in this cognitive state, the new SA guidelines state that, first, if the situation is very urgent, the system should present the information which has been processed in information prioritization and summarization by using affordance for direct perception. Because the driver has only a limited time, the system should judge which information can be prioritized and summarized, and then this information should be presented to assist with direct perception in the limited time available. For direct perception, the interface design should apply affordance. According to normal, affordance is “the perceived and actual properties of the thing, primarily those fundamental properties that determine just how the thing could possibly be used”. [34] Affordance provides strong hints about how things operate [34].

Second, the information should cover a broad range of situations but focus on what is most important. The driver cannot recognize the whole situation at a moment when the driver is in a high time pressure state because human activities compete with each other for the limited time available [35]. Moreover, cognitive studies have found that time pressure leads people to narrow the range of information they process [36]. Thus, when the driver is in a high time pressure state, if the aiding system presents situation information that the driver is not able to focus on, it helps to achieve SA.

- Low Time Pressure State

In the low time pressure state, the driver can readily perceive the situation but may understand it incorrectly when the transfer of information is delayed. If the driver’s cognitive state changes to a high time pressure state, it affects the ability to understand the situation. For example, if the navigation system notifies the driver too late that a right turn should be made now, the driver’s vehicle needs to change lanes very quickly. At that time, the driver’s cognitive state will change to a high time pressure state until the task has been completed.

To present information to a driver in this cognitive state, the new SA guidelines state that, first, the system should provide information proactively to avoid the driver entering a high time pressure cognitive state. Studies performed by Bader, Siegmund, and Woerndl indicated that the proactive aiding system is helpful and can provide effective assistance while driving [37]. If the information is provided proactively, the driver can take sufficient time to consider the situation, and this leads to a higher level of understanding and projection.

Second, the system should provide more detailed information to facilitate correct SA. Since the driver has sufficient time to respond to the situation, the system can provide more detailed information for easy comprehension and projection.

## 4. A Case Study for the Cognitive State-Aware SA Design Guidelines

### 4.1. Case Study Scenarios

**Figure 3:**
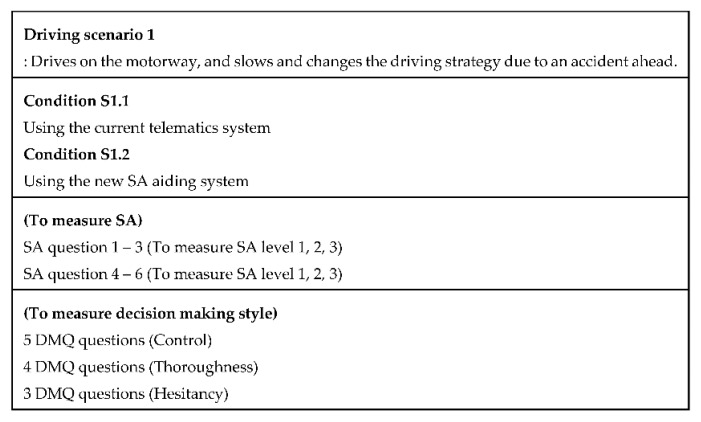
Descriptions of the first driving scenario (Source: Own creation).

**Figure 4:**
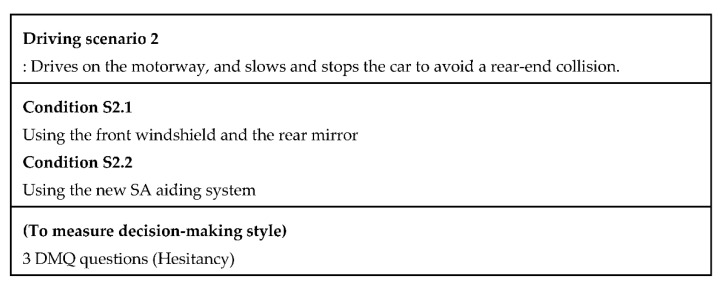
Descriptions of the second driving scenario (Source: Own creation).

We design two driving scenarios and four conditions to measure SA and decision-making style scores. In the first driving scenario, a participant drives on the motorway by interacting with one of the given systems and the participant receives situation information from the system that indicates that the driver needs to change the driving strategy due to an accident ahead (Figure 3). In the second driving scenario, the participant drives on the motorway, and then slows and stops the car to avoid a rear-end collision (Figure 4).

Scenario 1: The purpose is to measure the level of SA and decision making style. Two specific cognitive states were involved, the unfamiliar situation state and insufficient mental resources state. We designed a simulation that included an accident ahead. Changing the driving strategy is a factor that can induce an unfamiliar situation state. In addition, driving on a motorway leads to an insufficient mental state since the driving speed is faster than that of the local road. The study by Sugiono et al. also supported the fact that driving on the motorway induces a higher workload than driving on the urban and rural roads [38]. In the first driving scenario, the independent variables are measured using the current telematics system, and the new SA aiding system. The dependent variables are SA scores (Level 1, 2, and 3) and decision making scores (Control, Thoroughness, and Hesitancy). Thus, there are two conditions; condition S1.1 (as a control group) is driving with a Google map-like telematics system, which presents navigation route guidance, and condition S1.2 is driving with the new SA aiding system. The reason for choosing such a system is that people are familiar with this system in real life. The contents of the User Interface (UI) are the same, but the method of presenting information is different in the new system.

**Figure 5:**
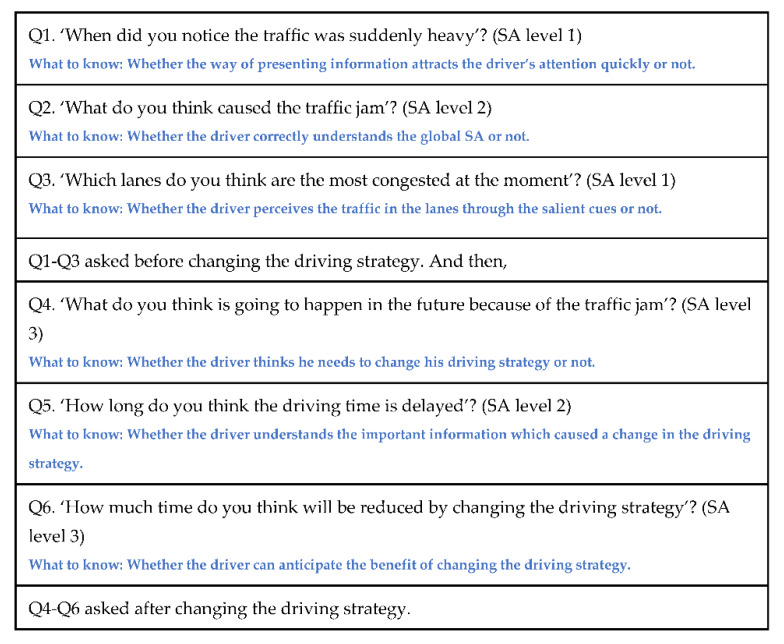
SA questions and what to know (Total of 6 questions) (Source: Own creation).

The method of presenting situation information in the SA aiding system is different in the new system. First, the new design presents information proactively to refocus the driver’s attention to the extent that it does not interfere with the driver’s primary task, giving them time to recall the proper knowledge necessary to update their mental model and to respond to the situation. Second, the new design divides a large amount of information to the driver little by little to give the driver sufficient time and to help the driver to understand which actions to perform. Third, to avoid increasing the driver’s workload and inducing an insufficient mental resources state, the new design presents information which has been processed in information prioritization and summarization as salient for direct perception to the extent that it does not interfere with the driver’s primary task. In order not to interfere with the driver’s primary tasks, the duration of the presented information should follow National Highway Traffic Safety Administration’s (NHTSA’s) driver distraction guidelines [39], which indicate that the mean duration of the driver’s eye glances away from the forward road scene should be shorter than or equal to 2 s for each interaction. In addition, the sum of all the interaction durations of the driver’s eye glances away from the forward road scene should be shorter than or equal to 12 s for completing the task at hand [39]. The new design will be illustrated in greater detail in Figures 10–12. We customized six SA questions for the first driving scenario (Figure 5) because when using SAGAT, the experimenter determines the queries to use for a particular experimental setting [3].

Figure 5 describes each SA question and what we wanted to learn from the answers. SA questions were used with SAGAT. Each SA question included multiple choices for the answer. Thus, if the participant chooses the correct choice, the participant gets one point.

**Figure 6:**
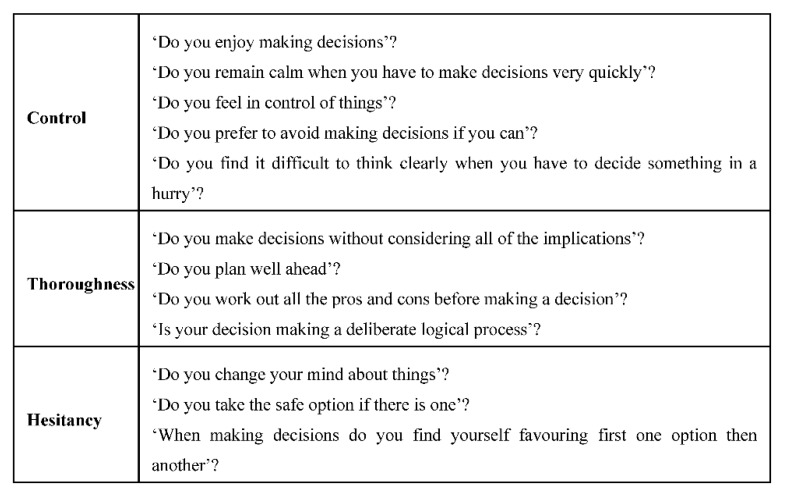
Decision-Making Questionnaire (DMQ) questions (Total of 12 questions) (Source: Own creation).

After we asked SA questions, we also asked DMQ questions. Among DMQ, we chose ‘Control’, ‘Hesitancy’, and ‘Thoroughness’ since those dimensions fit the driving situations. Figure 6 describes the questions in each dimension. The dimension ‘Control’ consisted of five questions with a six-point Likert scale. The maximum score of ‘Control’ is 30. The dimension ‘Thoroughness consisted of four questions with a six-point Likert scale. The maximum score of ‘Thoroughness’ is 24. The dimension ‘Hesitancy’ consisted of three questions with a six-point Likert scale. The maximum score of ‘Hesitancy’ is 18.

Scenario 2: The participant was asked to drive on the motorway (Figure 4). During driving, the participant recognizes a sudden traffic jam ahead. Thus, the participant needs to push a brake urgently and stop the car by carefully checking for other vehicles around the car. A high time pressure state was involved in the second driving scenario. We designed an emergency brake situation that required the car to stop urgently to induce a high time pressure state. The independent variables include using the new SA aiding system and using the front windshield and the rear-view mirror, and the dependent variable is the hesitancy score. Thus, there are two conditions. Condition S2.1 requires the participant to reduce speed when the participant recognizes the traffic jam with seeing the front of it. In addition, the participant is also required to stop the car carefully by using the rear-view mirror. Condition S2.2 uses the new SA system. Condition S2.1 is the control group, and the reason for choosing this system is that people are familiar with it because they have already used it in real life.

In a high time pressure state, the driver cannot understand the situation easily and quickly [3] because the driver cannot recall relevant knowledge from long-term memory quickly. In addition, a slow understanding of the situation induces a delay in decision making. In the second driving scenario, we measured ‘Hesitancy’ since we hypothesized that proactively presenting the whole situation would help to reduce the time needed to make decisions. We did not measure the SA score in the second driving scenario since proactively presenting information definitely causes earlier perception. The method of presenting the information by the SA aiding system is different from using the front windshield and the rear mirror. The SA aiding system presents information about the front and rear views with color blinking. In addition, the SA aiding system uses a voice interface to proactively alert the driver.

### 4.2. Descriptions of SA User Interfaces

- The Current Telematics System

We described the current telematics system which served as a control group. We then illustrated the features that the current system lacks in terms of cognitive state awareness.

**Figure 7:**
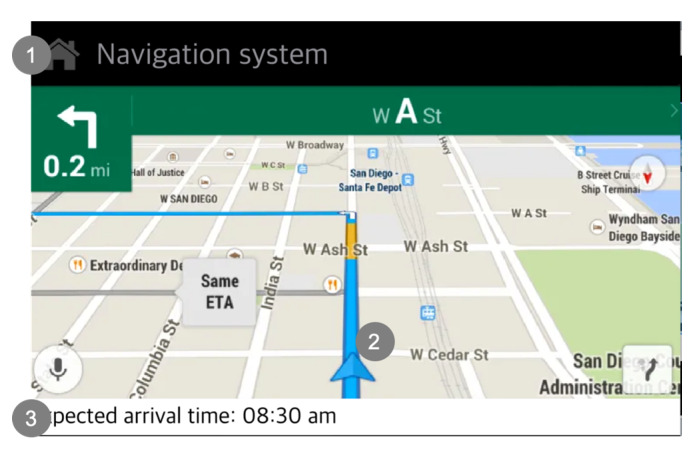
Descriptions of the main screen of the current telematics system (Source: Own creation): (**1**) The current telematics system presents the navigation route guidance; (**2**) It presents the navigation route guidance. It does not support [Global SA], but only navigation route guidance; and (**3**) It only presents expected arrival time, but do not present how long it takes to the destination (It does not support summarized information).

Scenario 1 (Condition S1.1): A current telematics system is our control group in condition S1.1 (Figure 7). The telematics system presents the same information but in a different way. The current telematics system only presents guidance about the navigation route, and it does not present information about the whole situation. When the system presents information related to time, it only provides expected arrival time. It does not support direct perception about how long it takes to reach the destination.

**Figure 8:**
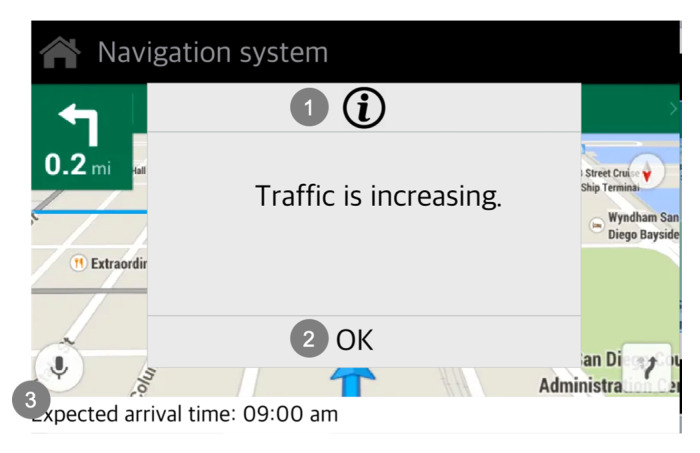
Descriptions of the pop-up screen of the current telematics system (Source: Own creation): (**1**) Through a pop-up, the telematics system informs the traffic info, however, it does not present information about the accident that occurred ahead, but only the part of the situation information presented; (**2**) It only supports a limited interaction by using a button; and (**3**) It does not support direct perception regarding the remaining time; the driver needs to calculate as the change of expected arrival time (No direct perception supported).

Figure 8 describes that the pop-up screen of the current telematics system used in condition S1.1. In this scenario, there is an accident that occurs ahead where the driver cannot see. The current telematics system informs the driver of the situation via a pop-up message, providing information about the disadvantages of the current driving choice. The current telematics system only presents traffic information without explaining the cause of traffic.

**Figure 9:**
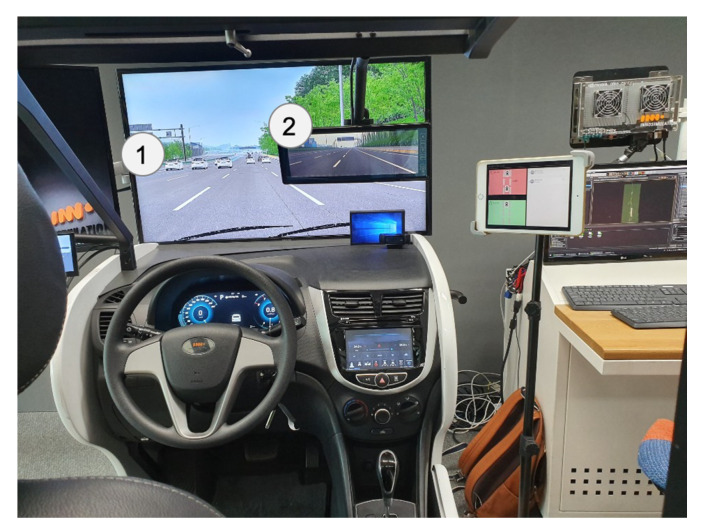
Descriptions of how the participant responds to the emergency braking situation (Source: Own creation): (**1**) the participants can use the frontal windshield since the telematics system does not support the function that informs frontal situation information and (**2**) the participant can recognize the rear situation through the rear camera.

Scenario 2 (Condition S2.1): Figure 9 describes how the participant responds to the emergency braking situation without the system.

This is used in condition S2.1. In this scenario, the driver is required to push the brake and stop the car since the traffic is suddenly heavy. There is no aid provided by the system since the current telematics system is not equipped with such a function. The participants were required to perform this manually using the frontal windshield and rear camera.

- The New SA Aiding System Prototype

We describe how the new cognitive state-aware SA design guidelines are applied to the prototype.

**Figure 10:**
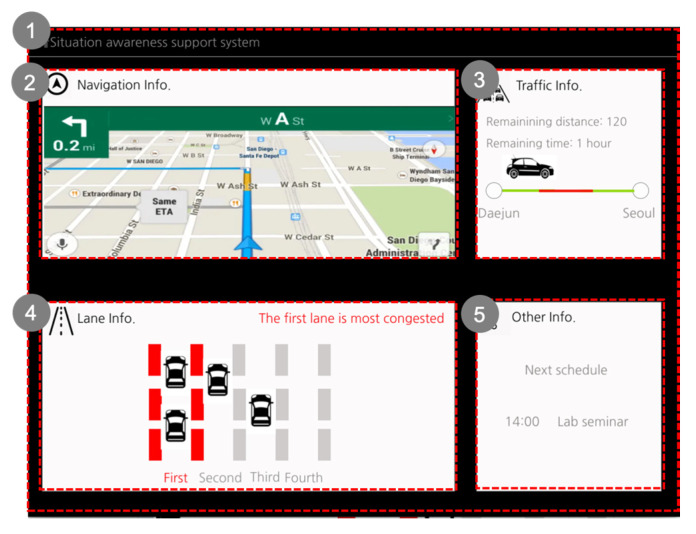
Descriptions of the main screen of the SA aiding system (Source: Own creation): (**1**) The main screen consists of four sections to provide global SA and high priority information for each section; (**2**) Navigation route guidance (No real data due to GPS disconnection); (**3**) Traffic information (it indicates traffic information on the route); (**4**) Lane information (The color highlights which lane is free or heavy); and (**5**) Other information (the personal schedule which can influence the driving strategy).

Scenario 1 (Condition S1.2): Two cognitive states: insufficient mental resource state and unfamiliar situation state are considered. Figure 10 describes the main screen of the new SA aiding system for the first driving scenario. The main screen was designed to support ‘insufficient mental resource state’. In the insufficient mental resource state, expected SA problems occur when the driver is unable to reflect deeply on the situation even though the driver detects a signal or cue because the situation is changing constantly, while the driver’s mental capacity is limited. The new SA design guidelines indicate that information can be better perceived via prioritization and summarization in a manner salient for direct perception. Because the driver is in an insufficient mental resource state, the driver cannot accept a large amount of information at a time. Thus, the driver should receive a small amount of information, and it should help the driver to project what will happen soon.

After consideration of the related SA errors which may occur in an insufficient mental resource state, we designed the main screen on the new SA aiding system as shown in Figure 10. The system main screen consists of several sections to help the driver’s driving strategy. Zhang et al. suggested that driving strategy refers to the combination of acceleration mode and velocity values chosen by the driver to traverse a given distance [40]. Thus, the main screen composed of four sections: navigation route, traffic information on the route, lane information, and other important information, such as personal schedule.

Compared to the current telematics system, the new SA aiding system presents essential and relevant information directly about the driving strategy to the destination. Navigation route information presents accurate information about a fastest route to the destination. Traffic information presents information about traffic along the whole route, which helps the driver recognize the time, traffic, and distance easily. The key difference between this system and the current telematics system is that the driver can quickly recognize where the traffic is heavy on the driving route. Lane information presents information about the traffic in the lanes, which helps the driver recognize which lane has less traffic. Although the telematics system does not provide information about traffic in the lanes, the new SA aiding system does provide it for effective driving. Other important information, such as the schedule, may influence the driving strategy. Thus, through four sections, the system presents more diverse information to expand the global SA and to influence the driving strategy to the destination.

In each section, the SA guidelines are applied. Thus, the prioritized situation information is filtered and presented, and this helps the driver recognize situation information with a lower cognitive load. Through color highlighting, the SA aiding system supports the driver’s direct perception of the traffic on both the route and the lanes.

**Figure 11:**
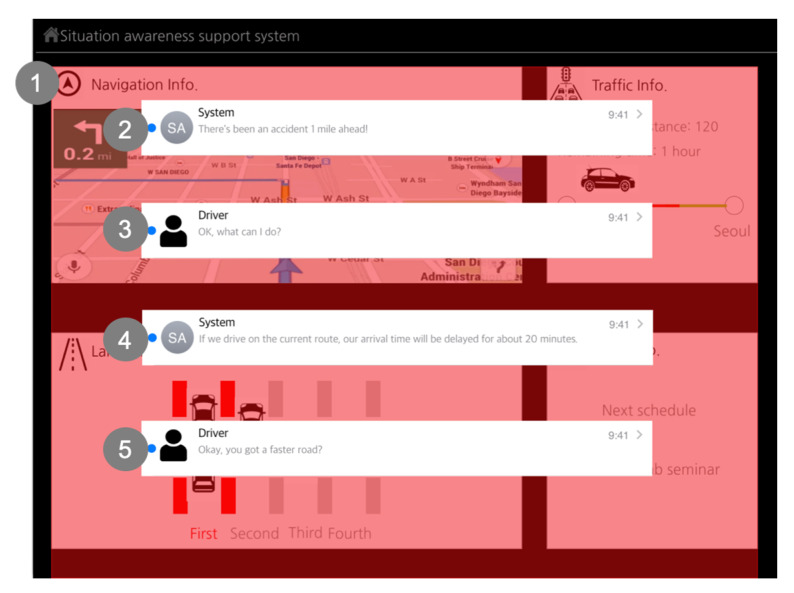
Descriptions of the interaction screen between the SA system and the participant (Source: Own creation): (**1**) It supports [Direct Perception]. The driver’s attention becomes arousal through proactively blinking red and green colors and it switches from driving to the situation information fast; and (**2**) it supports [Active Communication]. The driver can communicate actively with a system and it helps comprehension of the situation via voice interaction; (**3**) After the driver understands the situation, the driver can ask for more information which affects the projection; (**4**) Since the system tells the disadvantage of the current route, the driver can change the driving strategy; and (**5**) The driver checks the options to change the driving strategy.

The next screen (Figure 11) appears when a situation occurs which influences the driving strategy, which supports the driver when in an unfamiliar situation state. In an unfamiliar situation state, expected SA problems happen when the driver cannot understand the cue or alert even if they fail to observe it. In addition, understanding, projection, decision-making, and action are relatively slow or inappropriate in an unfamiliar situation because the driver may not have an appropriate mental model, and it takes time to relate to similar experiences or knowledge.

To correspond to the problem, the new SA design guidelines recommend presenting information proactively to refocus the driver’s attention, as well as giving the driver time to respond. In addition, it should help the driver to complete the required action by supporting comprehension and accurate projection. The driver can communicate with the SA aiding system step-by-step through two-way communication. Step by step means that the SA aiding system does not present all information at one time, but it presents a small amount of situation information in a specific order. It presents an alert first through color blinking and a voice message to help “Perception”. Next, it presents detailed situation information for “Comprehension”, and then it presents guides on how to act for “Projection”.

**Figure 12:**
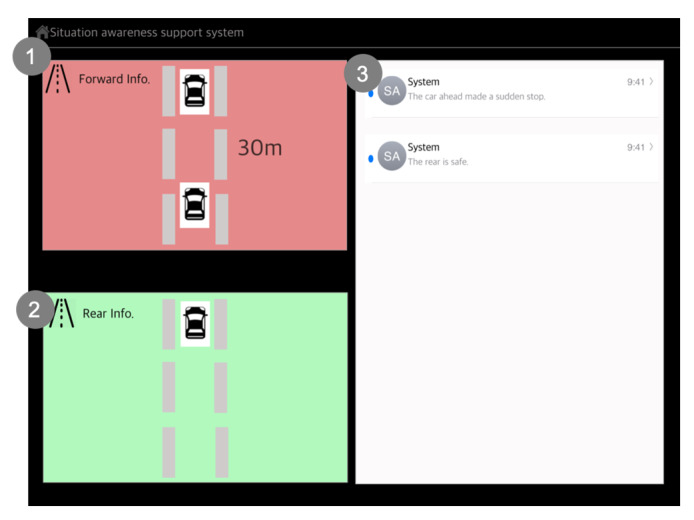
Descriptions of the interaction screen for a sudden braking situation (Source: Own creation): (**1**,**2**) The information covers a broad range of situations, but it focuses on the important. The driver’s attention becomes arousal through proactively blinking red and green colors and it switches from driving to the situation information fast [Direct Perception]; and (**3**) It supports [Active Communication]. The driver can communicate actively with a system and it can help comprehension of the situation.

Scenario 2 (Condition S2.2): Figure 12 describes how the new SA aiding system helps the driver in a sudden braking situation. Thus, the screen of Figure 12 presents situation information to a driver who is in a high time pressure state. During driving, there are multiple primary tasks, including controlling tasks like the steering wheel, accelerator, brake, and monitoring tasks, such as looking at the roads, other cars, or hazards. The driver should perform the above tasks concurrently, but human activities are in competition with each other for the limited time available [35].

Because human cognition is limited, if a driver concentrates on one particular task, the driver is forced to pay less attention to the other tasks.

In a high time pressure state, this symptom becomes severe. We assumed that controlling tasks and monitoring tasks compete more when there is a limited time. In addition, during monitoring tasks, the driver needed to monitor various areas using visual scanning to detect objects and events that might appear. When driving, the driver needs to monitor the forward, side, and rear view. During monitoring tasks, monitoring of each view is competing simultaneously for the driver’s attention. The driver cannot monitor all regions concurrently in a high time pressure state.

We hypothesized that if the driver is in a high time pressure state, the expected SA problems are that the driver can only focus on part of the phenomenon or task even though the driver also needs to focus on other elements. Because the driver has to complete the most pressing tasks in a limited time and while completing the task, the driver reacts to time pressures by narrowing down the number of alternatives [26]. We assumed that the driver cannot see forward and rear information concurrently, especially during the high time pressure state. The is because, at that moment, the driver’s cognitive process is complex, and it involves processes such as calculating the distance between the car being driven and that at the front, and the time required to stop the car. Thus, there is a risk that mistakes will be committed due to the urgency of the task.

To address these problems, the new SA design guidelines indicate that the SA aiding system should present situation information which covers a broad range of situations, while focusing on the important information. In addition, when the SA aiding system provides situation information, the system should filter the information to provide prioritized and summarized information. This will assist the driver’s direct perception since the driver has limited time to respond in a higher time pressure situation.

### 4.3. Participants and Materials

**Figure 13:**
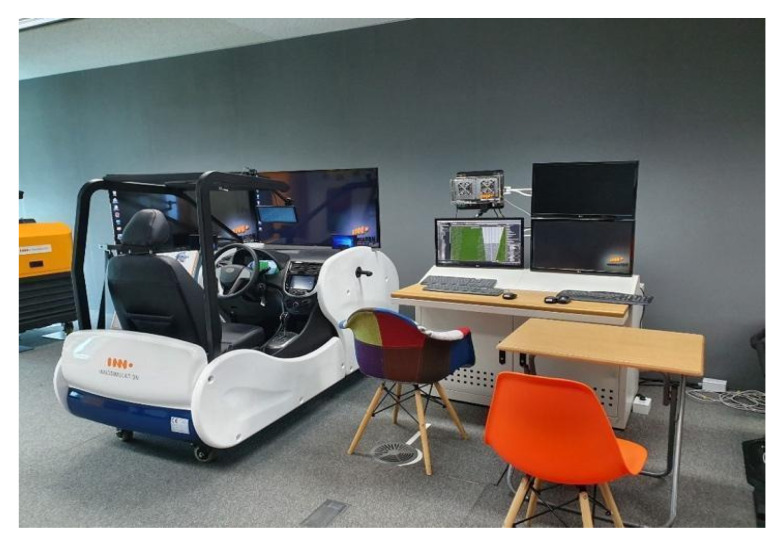
A driving simulator which can simulate various driving environments (Source: Own creation).

In total, 22 participants (19 males and 3 females) with driving licenses were approached by e-mail and personal contact and invited to participate in the study. They ranged in age from 22 to 49 years (mean age = 33 years). The average of their driving experiences was 9.14 years (SD = 6.80). The driving simulator’s displays presented front, side and rear views (Figure 13), and a cluster display indicated speed as in a real car. The main software used to control the driving simulator is SCANeR Studio version 1.8. The experimenter can change components of the driving scenario such as time of day or night, weather, number of cars and accidents, etc. The driving course was the Gyungbu motorway in Korea.

### 4.4. The Case Study Procedure

**Figure 14:**
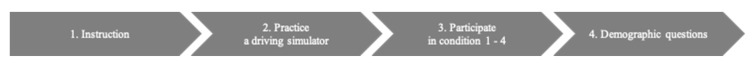
The case study procedure (Source: Own creation).

There are four stages in the case study (Figure 14). In the instruction stage, the participants were asked to listen to an explanation of the purpose of the case study and the procedures. During driving simulator practice, the participants were given 3–5 min to practice using a driving simulator in order to avoid possible sickness. In the participation stage, the participants needed to participate in two driving scenarios. During driving, the participants interacted with either system and relayed their respective situation information out loud. Then, the system paused, and the screen was changed to blank, and the participants were given a questionnaire that contained combined queries of SAGAT and DMQ. In the last stage, all the participants were asked to answer all demographic questions regarding gender, age, and driving experience.

## 5. Results

**Figure 15:**
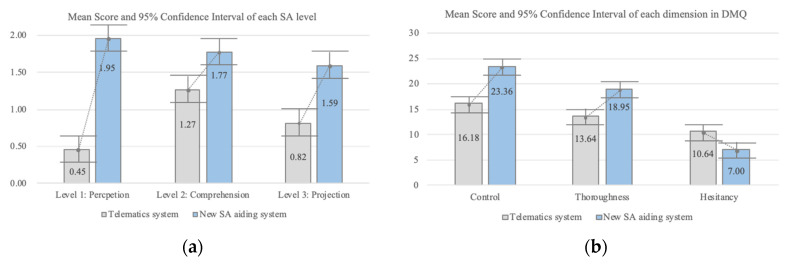
Mean Score and 95% Confidence Interval graph of SA and DMQ: (**a**) Mean score and 95% Confidence Interval of each SA level and (**b**) Mean score and 95% Confidence Interval of each dimension in DMQ (Source: Own creation).

The result of the case study was analyzed from two perspectives: SA and DMQ scores (Figure 15).

SA Scores: SA scores were measured and compared in conditions S1.1 and S1.2 in the first driving scenario. The SA score was based on six questions (two points for each level of SA) with a maximum possible score of 6. The mean score for the new SA aiding system was 5.22, and the mean score for the current telematics system was 2.54. There was a significant difference between conditions (F = 80.42; *p* < 0.01). The result confirms that the system that applied the new cognitive state-aware SA design guidelines conveyed situation information more effectively than the current telematics system, with significant differences between conditions at each level of situation awareness.

For level 1 SA (perception), the mean score for the new SA aiding system was 1.95 as compared to a mean score of 0.45 for the current telematics system (F = 162.19; *p* < 0.01), indicating a significant difference between the conditions for level 1 SA (Figure 15a). For level 2 SA (comprehension), the mean score of the new SA aiding system was 1.77 as compared to the current telematics system’s mean score of 1.27 (F = 10.29; *p* < 0.01). Although the F-value was relatively lower, this again indicates a significant difference between conditions. For level 3 SA (projection), the mean score of the system applying the new cognitive state-aware SA guidelines was 1.59 as compared to a mean score of 0.82 for the current telematics system (F = 14.84; *p* < 0.01), indicating a significant difference between conditions S1.1 and S1.2. Overall, these results indicate that the new cognitive state-aware SA design guidelines influenced level 1 SA (perception) more than the other levels of SA (comprehension and projection). We also analyzed the correlation between the driving experience and the total SA score (Table 3).

The results indicate that there are no significant correlations. The correlation score between the driving experience and the telematics system was 0.34 (*p* = 0.09; F = 3.12). The correlation score between the driving experience and the new SA aiding system was 0.26 (*p* = 0.29; F = 1.18).

DMQ Scores: We measured three dimensions of DMQ in the first driving scenario: “Control”, “Thoroughness”, and “Hesitancy.” Figure 15b indicates that these results confirm the differences in mean scores for each DMQ dimension. Figure 15b shows that using a system that applies the new SA design principles produces more effective decision making than the current telematics system.

**Figure 16:**
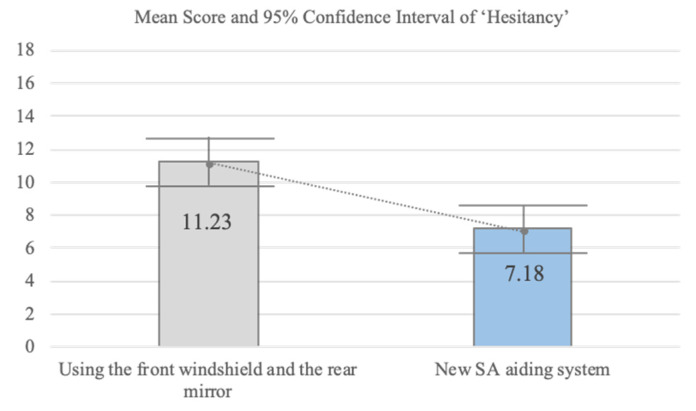
Mean Score and 95% Confidence Interval graph of ‘Hesitancy’ (Source: Own creation).

In relation to control, the mean score of the current telematics system was 16.18 and the mean score of the new SA aiding system was 23.36 (F = 40.64; *p* < 0.01), indicating a significant difference between conditions S1.1 and S1.2. In relation to thoroughness, the mean score for the telematics system (13.64) was lower than for the new SA aiding system (18.95) (F = 24.11; *p* < 0.01), indicating a significant difference between conditions S1.1 and S1.2. In relation to hesitancy, the mean score for the new SA aiding system was significantly lower than for the current telematics system (F = 13.79; *p* < 0.01). We also analyzed the correlation between the driving experience and each DMQ score (Table 4). The correlation score between the driving experience and each DMQ score of the telematics system: Control = −0.25 (*p* = 0.26; F = 1.32), Thoroughness = −0.14 (*p* = 0.53; F = 0.40), Hesitancy = −0.04 (*p* = 0.85; F = 0.04). The correlation score between the driving experience and each DMQ score of the new SA aiding system: Control = 0.19 (*p* = 0.39; F = 0.78), Thoroughness = −0.05 (*p* = 0.84; F = 0.04), Hesitancy = −0.18 (*p* = 0.42; F = 0.68). In the second driving scenario (conditions S2.1 and S2.2), participants were placed in a higher time pressure state, and hesitancy in decision making style was measured to validate the effectiveness of the new SA design principles. Figure 16 shows that hesitancy was significantly lower when using the new SA aiding system than when using the front and rear-view mirrors. The mean score for the new SA aiding system was 7.18 as compared to 11.23 when using mirrors (F = 15.27; *p* < 0.01). The correlation score between the driving experience and hesitancy rear score of the telematics system: Hesitancy Rear = −0.22 (*p* = 0.33; F = 1.02), the correlation score between the driving experience and hesitancy rear score of the new SA aiding system: Hesitancy Rear = −0.17 (*p* = 0.46; F = 0.57).

## 6. Discussion

Although Endsley’s SA principles are well-known, we showed that there is a lack of detailed guidelines for dealing with limited human cognitive capabilities. To address this issue, we proposed a new supplementary SA design guidelines based on cognitive states and developed a prototype SA aiding system to provide an example system that applied the cognitive state-aware SA guidelines. We tried to demonstrate how applying supplementary guidelines to the design of the SA aiding system could help drivers develop SA and improve their decision making. Thus, we compared the prototype to the current telematics system in two driving scenarios.

SA: In an insufficient mental resources state, the guideline that the system presents salient information, which has been processed according to information prioritization and summarization, was effective in enhancing SA. The UI was designed to convey the prioritized and summarized information about the traffic on the lane, it leads not only to the development of a participant’s global SA, but also an increase in level 1 SA. In an unfamiliar situation state, the system provided information to the driver little by little, and this was effective for allowing the driver to have sufficient time and to understand which actions to perform. It leads to both a higher level 2 and 3 SA, which correspond to comprehension and projection. In contrast, the telematics system only presented situation information via a pop-up message. The pop-up messages could not display step-by-step or complex messages without causing driver distraction.

Decision-Making: In an insufficient mental resources state, we assumed that the four sections of the main screen helped the driver feel control and thoroughness since the SA aiding system presented global SA regarding the situations which influence the driving strategy to the destination. In the unfamiliar situation state, presenting situation information step-by-step helped the driver to not feel hesitancy since the SA aiding system provided the driver with relevant situation information in an order corresponding to the level of SA. In a high time pressure state, the guideline states that the information should cover a broad range of situations, but focus on the important. Thus, we designed the SA aiding system to present emergency information consisting of the forward information and rear information necessary to avoid the collision. The new SA aiding system helped the participants to check all environments during a sudden braking situation. In addition, the SA aiding system proactively alerted the participants through voice interaction. The hesitancy score indicates that using the SA aiding system helps the participants to make a decision without hesitation. This means that proactively presenting situation information helps to mitigate the demands of the high time pressure state. In addition, green or red color blinking on the forward and rear information screens helps the participants to directly perceive the driving environment. It also reduces hesitancy in the driver’s decision-making.

Relationship with Driving Experience: Regarding the relationship between experience and SA, the research of Carretta, Perry, and Ree indicated flying experience is one of the important attributes affecting the SA in aviation [41]. However, in this research, it turned out there was no significant relationship between driving experience and SA and decision-making style. Because the training intensity is different between the pilot and the driver. The pilot is required to be trained for a longer time and it leads to more high-level skills to achieve SA. The research of Underwood, Ngai, and Underwood also indicated that there is no significant relationship between driving experience and situation awareness [42]. In addition, the driving scenarios in the case study are not difficult, every participant could easily perceive and interpret the situation information from both systems.

Limitations: Our SA design guidelines will help the designer to consider various cognitive states when designing new SA aiding systems. Thus, the designer can analyses several SA errors related to cognitive states, and then they can design the SA aiding system interface while considering various cognitive states. We conducted a case study to verify the effectiveness of the guidelines. However, the system interface in the case study was designed based on the current driving strategy. In the future, more complex situation information which affects the driving strategy will be developed, and there is no way for us to know right now what this information will include. There are limitations of using SAGAT in the simulator environment. Because the system should be frozen and blank during answering, the answering relies on the participant’s memory [43]. In addition, SAGAT and DMQ measured the subjective status of the participants, and the results might be different from the driver’s real driving action. Although there are some limitations, these methods have been widely used in various scenarios (e.g., aviation, air traffic control, and driving) and were validated to measure SA score and decision-making style [3,10]. The methods of overt communication and alerts should be used with caution. Generally, overt communication and alerts are used to attract the driver’s attention. However, they might attract too much attention, and interfere with the primary driving tasks.

There are several directions for future work. First, we need to discover how the SA aiding system can measure the human cognitive state correctly in a reasonable time frame. There are some accurate ways to physiologically measure the cognitive state of a driver, such as eye-tracking or electroencephalography (EEG) [44]. Second, further research is required to understand how the new SA guidelines can be applied in domains other than driving. We applied the new SA guidelines in only the driving environment. Third, we need to provide ways in which the design applies to other cognitive states. In this research, we only provided limited cases of three cognitive states in which the guidelines can be used. Future work should provide ways in which the guidelines can be used in other cognitive states. Fourth, we need to provide methods to optimize various attributes that can be used to decide the driver’s cognitive state. The smart vehicle will be able to monitor the driver’s cognitive states with vehicle-based data, behavioral state-based data, and physiological-based data [43,45]. We assumed that the advanced aiding system will be able to quantify specific information about each cognitive state, as well as provide the binary coding for each attribute. With this assumption, we can expand the attributes related to human cognitive states to an n-dimensional decision space. Further, it is possible to formulate the problems of information presentation and user interactions as combinatorial optimization [46]. Lastly, some unexpected situation information will appear as technology develops further. Hence, we need to discover more information about unknown situations for the future system.

## 7. Conclusions

Our goal was to determine how to provide various complicated situation information related to the driving strategy to the driver while minimizing additional cognitive burdens. Our analysis of previous SA design principles revealed that supplementary SA design guidelines are needed because the existing principles lack detailed guidelines on how to apply the principles according to diverse cognitive states. We proposed new supplementary SA design guidelines that discuss relevant SA problems and possible solutions in each cognitive state. To demonstrate the usefulness of our design guidelines, we conducted a case study. We hypothesized that our new SA aiding system would allow participants to achieve higher SA and support effective decision making. Our results indicated that the new system was able to enhance the driver’s SA significantly more than the current telematics system. In the aspect of decision-making, the new system was also helpful in the unfamiliar situation state, insufficient mental resource state, and high time pressure state. In future work, it would be interesting to consider dynamically changing cognitive states and propose design principles for more adaptive and intelligent support systems. In addition, we will find out the way of optimization for the system that judges the driver’s cognitive state while driving. Furthermore, it is important to explore how our supplementary SA design guidelines can be applicable to other domains such as aviation. We also need to expand our guidelines so that they can be used in all cognitive states.

## Figures and Tables

**Table 1:** SA principles suggested by Endsley [3].

No.	Principle	Descriptions
1	Organize information around goals	Information should be organized according to the main objectives of the operator rather than expressed in a technology-oriented manner
2	Present Level 2 information directly—support comprehension	The degree to which integrated information satisfies level 2 SA will positively influence SA
3	Provide assistance for Level 3 situation awareness projections	The operator is required to develop a proper mental model to make projections about future states
4	Support global situation awareness	The operator should have a high-level overview of the situation related to the goals
5	Support trade-offs between goal-driven and data-driven processing	It deals with the tension between top-down processing and bottom-up processing when designing a system.
6	Make critical cues for schema activation salient	Activate cognitive functions by offering saliency in user interactions
7	Take advantage of parallel processing capabilities	The operator can share attention in SA
8	Use information filtering carefully	Cognitive overload could hinder situation awareness, and filtering can lower the cognitive workload

**Table 2:** New Situation Awareness Design Principles based on the Cognitive States (Source: Own creation).

Cognitive State/Category	SA Problems	Guidelines
Focused attention state (Attention)	The driver’s attention is too narrow to perceive the overall situation around the event (perception error).	1. The system provides global SA information for effective driving.
Inattentional blindness state (Attention)	The driver cannot recognize a cue even if he/she sees it (perception error).	1. The system uses overt communication to attract the driver’s attention.
Unfamiliar situation state (Mental Model)	The driver cannot understand the cue or alert even if he sees it (comprehension error). Understanding, projection, decision-making, and action are relatively slow or inappropriate because the driver lacks an appropriate mental model, and it takes time to retrieve similar experiences or knowledge.	1. The system presents information proactively to refocus the driver’s attention to the extent that it does not interfere with the driver’s primary task, giving them time to recall the proper knowledge to update the mental model and respond to the situation. 2. The system divides a large amount of information and provides it to the driver little by little to give the driver sufficient time and to help the driver to understand actions to perform.
Familiar situation state (Mental Model)	In a familiar and an anticipated situation, the driver performs a skill-based behavior. At this level, he may commit skill-based errors (slips or lapses) (action error) since the driver over-relies on the old version of the information in the model (projection error).	1. The presented information needs to help the driver to update the mental model with new information to avoid over-rely on the old version of the information in the mental model and to project accurately the future situation. 2. The aiding system should indicate appropriate actions sequentially at the appropriate time to help the driver execute these actions.
Insufficient mental resources state (Workload)	The driver is unable to reflect deeply on the situation even though he/she detects a signal or cue because the situation is changing constantly (errors of perception, comprehension, projection, and decision).	1. The system presents information which processed in information prioritization and summarization as salient for direct perception to the extent that it does not interfere with the driver’s primary task to not increase workload.
Sufficient mental resources state (Workload)	The driver is able to consider the situation in detail to understand it correctly. The driver’s workload may be increased depending on the amount of information given at a time, and the timing of presenting the information since the driver is focusing on performing the primary tasks (It leads the driver to enter an insufficient mental resource state).	1. The system can present more detailed information to enhance Level 2 and Level 3 SA, but information should be configured and provided to avoid increasing the workload too much. 2. The system emphasizes information that needs to be known in the moment the extent that it does not interfere with the driver’s primary task.
High time pressure state (Human memory)	The driver can only focus on part of the phenomenon or task and does not care that he/she needs to focus on other elements. There is a risk that mistakes will be committed due to the urgency of the task (errors of perception, comprehension, projection, decision, and action).	1. If the situation is very urgent, the system presents the information which is processed in information prioritization and summarization by using affordance for direct perception. 2. The information covers a broad range of situations, but focuses on those that are most important.
Low time pressure state (Human memory)	Since the driver is focusing on performing the primary tasks, the driver may enter a high time pressure state if the transfer of information is delayed. The driver can readily perceive the situation but may understand it incorrectly (errors of comprehension, projection, decision, and action).	1. The system provides information proactively to avoid a high time pressure cognitive. 2. Provide more detailed information for correct situation awareness.

**Table 3:** The statistical analysis of SA scores: One-way ANOVA and the correlation (Source: Own creation).

	Correlation	F-Value	*p*-Value
One-Way ANOVA: Total SA score	-	80.42	<0.01
One-Way ANOVA: SA Level 1	-	162.19	<0.01
One-Way ANOVA: SA Level 2	-	10.29	<0.01
One-Way ANOVA: SA Level 3	-	14.84	<0.01
Correlation: driving experience and the telematics system	0.34	3.12	0.09
Correlation: driving experience and the new SA aiding system	0.26	1.18	0.29

**Table 4:** The statistical analysis of DMQ scores: One-way ANOVA and the correlation (Source: Own creation).

	Correlation	F-Value	*p*-Value
One-Way ANOVA: Control	-	40.64	<0.01
One-Way ANOVA: Thoroughness	-	24.11	<0.01
One-Way ANOVA: Hesitancy	-	13.79	<0.01
One-Way ANOVA: Hesitancy (Rear)	-	15.27	<0.01
Correlation: driving experience and the telematics system (Control)	−0.25	1.32	0.26
Correlation: driving experience and the telematics system (Thoroughness)	−0.14	0.40	0.53
Correlation: driving experience and the telematics system (Hesitancy)	−0.04	0.04	0.85
Correlation: driving experience and the telematics system (Hesitancy_Rear)	−0.22	1.02	0.33
Correlation: driving experience and the SA aiding system (Control)	0.19	0.78	0.39
Correlation: driving experience and the SA aiding system (Thoroughness)	−0.05	0.04	0.84
Correlation: driving experience and the SA aiding system (Hesitancy)	−0.18	0.68	0.42
Correlation: driving experience and the SA aiding system (Hesitancy_Rear)	−0.17	0.57	0.46
